# Supplementary material for: The Dual Prey-Inactivation Strategy of Spiders—In-Depth Venomic Analysis of Cupiennius salei
Source: Toxins (Basel). 2019 Mar 19;11(3):167. doi: 10.3390/toxins11030167 (PMC6468893; doi:10.3390/toxins11030167)
Supplement: Supplementary file 1 [file toxins-11-00167-s001.zip › Supplementary Fig S5/Supplementary Fig S5.pdf]

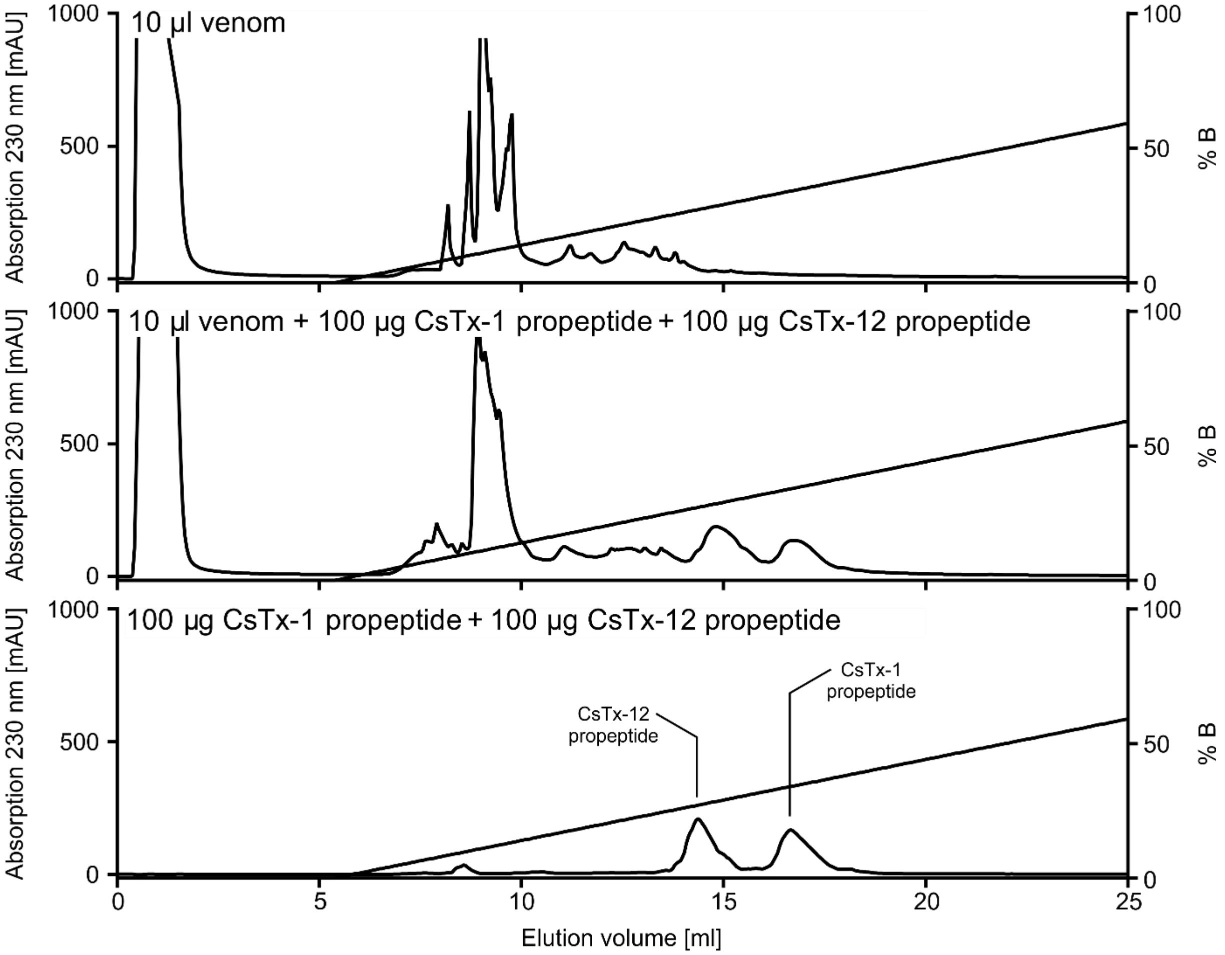

Supplementary Fig S5. Anion exchange of *C. salei* venom and synthetic propeptides of the neurotoxins CsTx-1 (EIEDDFLEDESFEAEDIIPFFENEQA) and CsTx-12 (ETEDDFLEDESFEADDVIPFLAREQVR).

The chromatography was performed on a MiniQ PE 4.6/50 strong anion exchange column (GE Healthcare) using 20 mM TRIS-HCl pH 9.5 as mobile phase. Analytes were eluted by applying a gradient of 0 M to 1 M NaCl in 40 min at a flow rate of 0.8 ml /min. In case intact propeptides should be present in the venom, the amount of CsTx-1 propeptide in 10 µl venom was estimated to 44 – 103 µg according to the venom concentration of CsTx-1 (1.4 – 3.3 mM).
